# Reforming the Portuguese mental health system: an incentive-based approach

**DOI:** 10.1186/s13033-018-0204-4

**Published:** 2018-05-30

**Authors:** Julian Perelman, Pedro Chaves, José Miguel Caldas de Almeida, Maria Ana Matias

**Affiliations:** 1 Escola Nacional de Saúde Pública, Universidade NOVA de Lisboa, Avenida Padre Cruz, 1600-560 Lisbon, Portugal; 2 Centro de Investigação em Saúde Publica, Escola Nacional de Saúde Pública, Avenida Padre Cruz, 1600-560 Lisbon, Portugal; 3 NOVA Medical School, Campus Sant’Ana, Pólo de Investigação, NMS, UNL, Rua do Instituto, Bacteriológico, no 5, 1150-082 Lisbon, Portugal; 4 Nova School of Business and Economics, Universidade NOVA de Lisboa, Campus de Campolide, 1099-032 Lisbon, Portugal

**Keywords:** Innovative payment, Mental health, Access, Primary care

## Abstract

**Background:**

To promote an effective mental health system, the World Health Organization recommends the involvement of primary care in prevention and treatment of mild diseases and community-based care for serious mental illnesses. Despite a prevalence of lifetime mental health disorders above 30%, Portugal is failing to achieve such recommendations. It was argued that this failure is partly due to inadequate financing mechanisms of mental health care providers. This study proposes an innovative payment model for mental health providers oriented toward incentivising best practices.

**Methods:**

We performed a comprehensive review of healthcare providers’ payment schemes and their related incentives, and a narrative review of best practices in mental health prevention and care. We designed an alternative payment model, on the basis of the literature, and then we presented it individually, through face-to-face interviews, to a panel of 22 experts with different backgrounds and experience, and from southern and northern Portuguese regions, asking them to comment on the model and provide suggestions. Then, after a first round of interviews, we revised our model, which we presented to experts again for their approval, and provide new suggestions and comments, if deemed necessary. This approach is close to what is generally known as the Delphi technique, although it was not applied in a rigid way.

**Results:**

We designed a four-dimension model that focused on (i) the prevention of mental disorders early in life; (ii) the detection of mental disorders in childhood and adolescence; (iii) the implementation of a collaborative stepped care model for depression; and (iv) the integrated community-based care for patients with serious mental illnesses. First, we recommend a bundled payment to primary care practices for the follow-up of children with special needs or at risk under 2 years of age. Second, we propose a pay-for-performance scheme for all primary care practices, based on the number of users under 18 years old who are provided with check-up consultations. Third, we propose a pay-for-performance scheme for all primary care practices, based on the implementation of collaborative stepped care for depression. Finally, we propose a value-based risk-adjusted bundled payment for patients with serious mental illness.

**Conclusions:**

The implementation of evidence-based best practices in mental health needs to be supported by adequate payment mechanisms. Our study shows that mental health experts, including decision makers, agree with using economic tools to support best practices, which were also consensual.

## Background

To promote an effective mental health (MH) system, the World Health Organization (WHO) has made several recommendations, namely, a larger involvement of primary healthcare (PHC) in prevention and treatment of mild diseases, community-based care for serious mental illnesses (SMI), more integrated care, better access to care, and less discrimination [1]. An evaluation of the Portuguese mental health plan carried out in 2017 stated that Portugal is failing to achieve such recommendations [2]. The Portuguese mental health system is essentially centered around inpatient stays and emergency consultations, which consume more than 80% of the resources, coupled with an insufficient provision of community-based services [3]. A cross-country comparison has shown that Portugal is below other European countries in terms of development of community-based mental health centers and mental health teams [3].

These weaknesses are especially worrisome when considering that the prevalence of lifetime mental disorders is above 30% [4], that MH disorders represent 11.7% of disease-adjusted life years lost, and that Portugal experiences a high prevalence of depression (7.9%), anxiety (16.5%), impulse disorders (3.5%), and substance abuse (1.6%) in comparison with other European countries [4].

Several ambitious and evidence-based plans have been proposed over the last decades, but none of them has been able to convincingly tackle these issues. We documented, in a previous contribution [5], that this failure was partly due to the inadequate payment mechanisms of Portuguese MH care providers, which did not encourage best practices. Among these mechanisms we highlighted the volume-based hospital financing system, which does not encourage the continuity of care or community-based interventions; and the capitation-based model for PHC, which favors long lists and short consultations, completed by a pay-for-performance (P4P) scheme that does not include a single MH indicator.

Based on this perspective this study designs a new payment model for MH care providers in Portugal, focusing on the prevention and detection of MH disorders early in life, on the treatment of moderate depression in PHC, and on the community-based follow-up of SMI. The prevention and detection dimensions were first selected as major issues because of the large burden of mental disease in Portugal, in comparison with neighbor countries: for example, the 2017 Global Burden of Disease study indicates that major depressive disorders represented the third cause of years lived with disability in Portugal, 40% than predicted according to the country’s socio-demographic context, while it is the fifth cause in Western Europe, 10% higher than predicted [6]. The second reason for selection was the extreme weakness of mental public health in the country. As mentioned in the 2017 evaluation of the National mental health plan, “so far, Portugal has no integrated strategy for promotion and prevention in mental health” (p. 57) [2]. In regard to treatment of moderate depression in PHC, the WHO “Mission to assess the progress of the mental health reforms in Portugal” mentioned that “unless primary care services can treat the large minority of people with anxiety and depression, specialist services will be paralysed due to the demand, unable to focus on people with severe and ongoing needs” (p. 9) [7]. The same mission observed that “the financing system has created unintentional disincentives to establish community based services, rewarding hospital admissions and medical interventions” (p. 9). We further detail the rationale for selecting these dimension as priorities for reforming the payment system.

### Conceptual background

In the health economics literature, the physician (the agent) is viewed as making decisions on behalf of the patient (the principal), because he has more knowledge and information about diagnoses and treatments. However, the physician is rarely a perfect agent for the patient because he also cares about his own interests (income, leisure time, reputation, etc.). The physician’s objectives of patient well-being and own interest may conflict, which may result in the physician not always making the best decisions for the patient. This agency problem exists because of the impossibility for the patient to adequately monitor the physician’s effort and competence due to lack of knowledge and information, and the uncertainty surrounding treatments’ outcomes. The fact that patients lack information about their MH disease and possible treatments is especially acute because of the stigma surrounding these diseases, which inhibits open discussions and information search, while the uncertainty about treatments’ effectiveness is greater than in other clinical domains. These difficulties are amplified by the physicians’ own lack of information and knowledge for MH. For example, a focus group with general practitioners (GPs), conducted in the UK, observed that “serious mental illness (is) too specialized for routine primary care and felt they lacked sufficient skills and knowledge” [8]. Payment mechanisms are of particular importance to align physicians’ objectives of patient health and own well-being.

All traditional payment mechanisms have advantages and drawbacks. The fixed salary avoids incentives to discriminate against patients but limits the physicians’ motivation, introducing a risk of lower quality. Fee-for-service (FFS) motivates physicians to increase the volume of care, but may encourage an excess provision, leading to higher expenditures. Capitation, which reimburses practices on the basis of a list of potential users, promotes efficient use of resources but may lead to selecting the healthiest users, and to under-provision. The bundled payment, which reimburses providers for treating diagnosed patients for a given period regardless of services provided, creates incentives similar to those in capitation, except that it does not encourage the selection of healthy patients because it finances patients with a given disease. Finally, P4P rewards high quality care but may cultivate a practice centered exclusively on indicators, and the selection of patients who are more likely to help attain the targets. Let us mention also that FFS is more trusted by patients than other payment models, because they feel that under FFS physicians put the patients’ health and well-being above cost considerations [9].

Internationally, alternative reimbursement models have been tested in MH, with limited success. In the United States (US), the “Colorado Medicaid Capitation” replaced the traditional FFS system in 1995, which led to a reduction in the use of more complex resource-consuming services and lower expenditures [10], a greater integration of services [11], consultations replacing inpatient stays among youths, but no change in prevention [12]. The per case payment system, using Diagnosis Related Groups (DRG), was demonstrated to reduce institutionalization of SMI [13] but increased hospital debts, possibly because of the inadequacy of DRG as a classification system for MH, which are more oriented to short acute stays than long-term uncertain ones [14]. In Austria the creation of specific categories for MH allowed hospitals to cover their costs while increasing community-based care.

Finally, in the UK, characterized by an NHS with strong similarities to the Portuguese one, a payment per activity was implemented based on Healthcare Resources Groups (HRG). However, it was observed that this payment model offered few incentives to MH providers to respond efficiently to MH needs [15], so that episode-based payments were introduced, based on *Mental Health Clusters*. These clusters group patients into 21 categories, according to their needs, and providers are paid a fixed amount for each treatment period according to the patient’s cluster. Jacobs, Chalkley [15] analyzed this payment model, showing a high variation between providers in terms of costs, treatments, and lengths of stay within clusters, making the adequate pricing and services of each cluster difficult. These authors concluded that the payment should not be abandoned, as it was the most adequate for MH treatment, but that clusters should be revised in order to make them more homogenous.

To summarize, theory suggests using payment systems that combine various reimbursement schemes in order to attenuate their weaknesses, while the evidence is poorly conclusive about which system functions best in MH. Hence, our proposal is more grounded on theoretical considerations, adopting the following options:

1. When there was evidence that a specific service was a good practice, we opted to encourage it specifically through FFS.
2. Capitation and bundled payments were favored because they encourage efficiency, continuity of care, and prevention, but we completed these schemes with P4P in order to limit the risk of under-provision.

## Methods

Our goal was to create payment mechanisms that encourage the evidence-based best practices in mental health, not to define these best practices. This is why we performed a narrative review of the literature, in the four selected domains of action, to identify the best practices with proven effectiveness and cost-effectiveness. The option for a non-systematic review was guided by the fact that best practices have long been identified in systematic reviews, and reported in national and international guidelines, so that a duplication of this task was not deemed necessary. Namely, we used as reference, along the study, the book published by the European observatory on health systems and policies, “Mental health policy and practice across Europe” [16]; the chapters 8 (promotion of mental health and prevention of mental health disorders), 9 (common problems in primary care), and 10 (the balance between hospital and community-based mental care) were particularly used as references to identify best practices.

Thereafter, we elaborated payment mechanisms, which we further presented to a large panel of experts in the field, who had the opportunity to comment on the proposal and make suggestions. We interviewed 22 experts with different backgrounds and experience, and from southern and northern Portuguese regions. The list of experts included ten psychiatrists, four hospital managers with an economics background, two psychologists, two nurses, one hospital manager with a health science background, one social assistant, one public health specialist, and one GP. There were 13 men and 9 women, and the average experience as professional in the area was 22 years (ranging from 3 to 40) (see the list in Table 1).

**Table 1:** Characterization of experts

#	Profession	Sex	Experience (years)	Region
1	Biologist, hospital manager	M	11	North
2	Psychiatrist	M	37	South
3	Economist, hospital manager	M	18	South
4	Psychiatrist, hospital manager	M	36	North
5	Psychiatrist	M	39	North
6	Nurse	F	39	South
7	Psychiatrist	F	3	South
8	Psychologist	F	29	South
9	Public health physician	F	4	South
10	Psychiatrist	M	3	South
11	Economist, hospital manager	F	30	South
12	Psychiatrist	M	24	South
13	Psychiatrist	M	40	South
14	Psychiatrist	F	3	South
15	Nurse	M	14	South
16	Psychiatrist	M	35	South
17	Social assistant	F	25	South
18	Economist, hospital manager	F	10	North
19	Psychologist	M	19	South
20	Economist	M	11	South
21	Psychiatrist	F	10	North
22	GP	M	42	South

Our study cannot be considered as a qualitative analysis in a traditional way, which was beyond our scope and competences. However, we proceeded in a way that is close to the Delphi technique, with two rounds, as follows. We designed an alternative payment model, on the basis of the literature, and then we presented it individually, through face-to-face interviews, to the panel of experts, asking them to comment on the model and provide suggestions. Then, after a first round of individual interviews, and a collection of highly important and numerous comments and suggestions, we revised our model, which we presented to experts again for their approval, individually, and provide new suggestions and comments, if deemed necessary. The consultation rounds occurred between 29 February 2016 and 18 March 2016.

This approach is indeed close to what is generally known as the Delphi technique, although it was not applied in a rigid way, and our objective was more about improving our initial model by obtaining new ideas and measuring its feasibility in the Portuguese context, than to make it fully consensual (contrary to the principle of the Delphi technique, which aims at reaching consensus by way of statistical analysis [17]). Indeed, there was no explicit method to reach a consensus between experts, since they were interviewed individually, and had not the opportunity to see and comment on other experts’ suggestions. This is why our paper also does not display results of the expert panel. The results of the final model, which derive from our literature review and the inputs from experts, are reported.

## Results

We detail here the four dimensions of the proposal, describing the rationale for choosing one as a priority; the type of intervention that we chose to encourage, and why; and the proposed payment mechanism. The final proposals for each dimension are summarized in Table 2.

**Table 2:** Proposed model for MH providers’ financing

Dimension	Proposal for financing	Implementation aspects
1. Prevention early in life	Bundled payment to the PC team for the follow-up of children at risk or with special needs during the two first years of life	1. Children registration on a central platform, including information/justification for being considered at risk or with special needs, using a diagnosis evaluation grid 2. Presence of a psychologist available for consultation in PHC practices
2. Early detection of mental health disorders	1. Adding an indicator in the P4P scheme for PHC practices, namely the “percentage of users in the key-ages of the PNSIJ who have effectively attended the vigilance consultations, according to the diagnosis evaluation grid” 2. Payment of an additional fee to GPs for each follow-up consultation including MH evaluation, using the diagnosis evaluation grid	The diagnosis evaluation grid must be subject to public discussion, revised, and subject to a large approval by GPs. The current grid, defined by the PNSIJ, includes several mental health recommendations for children and adolescents, related to emotional and behavioral disorders, psycho-affective and social development, and environment safety
3. Stepped collaborative model for depression	1. Adding an indicator in the P4P scheme for PHC practices, namely the “Proportion of users with depression whose condition has been diagnosed with PHQ-9 and treatment has been initiated in the adequate phase of the collaborative stepped care model” 2. Payment of a fixed monthly fee to compensate these reference physicians for the extra work	1. Nomination of a reference GP in the PHC team and a reference psychiatrist in the specialised MH team of catchment area, to enhance the collaboration between primary and specialised care 2. Presence of a psychologist available for consultation in PHC practices
4. Integrated community-based care for SMI patients	1. Implementation of a per period payment, according to which the hospital receives an annual payment for each patient registered with SMI, covering all healthcare services 2. The payment is completed by a P4P component A bonus (resp. penalty) for the hospitals in the lowest (resp. highest) decile of the distribution in terms of inpatient stays A bonus (resp. penalty) for the hospitals in the lowest (resp. highest) decile of the distribution in terms of post-discharge consultations up to 30 days after discharge A budget penalty in case the hospital does not contribute and update a national registry of SMI, specifically created within this new payment model	1. The payment is attributed to the MH department, which has full autonomy and responsibility in managing funds, being the residual claimant 2. The MH department disposes of community-based MH teams, with protocols with PHC practices, residential units, patients and families associations, rehabilitation units, nursing homes, social services, and local authorities

### Prevention early in life

#### Rationale

There is vast evidence that early life adversities affect health in the long run [18]. This is particularly true for MH. Kessler, McLaughlin [19] estimate that parental MH disorders, parental criminality, family violence, and physical or sexual abuse, are all related to a higher likelihood of MH disorders during childhood, adolescence, and adulthood. Interventions early in life in socially deprived contexts have also been demonstrated to be highly effective in preventing physical and mental illnesses [20].

#### Intervention

The Portuguese National Plan for Child and Youth Health (PNSIJ) acknowledges this point, suggesting that “(…) it is crucial to evaluate: the adaptation to pregnancy; the emotional status of the mother; psychosocial factors” [21]. The text mentions, “The evaluation of the family dynamic should be a concern for the PHC team at each contact with the child/youth/family. During the first year of life, special attention should be devoted to the emotional status of the mother (due to the risk of post-partum depression), referring to the identified cases that may interfere in the child’s development”. The plan suggests personalized care for children at risk or special needs, with a higher frequency of consultations, and the possibility of at-home visits. These visits have been proven to be effective in avoiding MH disorders later in life [22, 23].

These proposed guidelines seem to represent an adequate response, but their implementation has been limited by the insufficient human resources and by the absence of a clear signal and compensation to PHC teams for whom early prevention of MH disorders should be a priority.

#### Payment model

We propose the creation of a bundled payment to the PHC team for the follow-up of children at risk or with special needs during the two first years of life, with the registration of these children on a central platform, including information/justification for these children being considered at risk or with special needs, on the basis of a diagnosis evaluation grid [24]. The presence of a psychologist available for consultation in PHC practices is also recommended (he/she does not need to be physically present full time, being preferably part of a specialized MH team).

### Early detection of mental health disorders

#### Rationale

Kessler, Berglund [25] observed, on the basis of a cohort, that half of MH disorders (Diagnostic and Statistical Manual of Mental Disorders, 4th Edition; DSM-IV) have their onset before 14 years old, and 75% before 24 years old. This study also observed that the median age of onset of anxiety and impulse disorders was 11 years old. However, Burnett-Zeigler, Walton [26] concluded that only a third of adolescents attending a PHC consultation receive a psychological evaluation.

#### Intervention

PHC practices are the best setting to tackle this issue. In Portugal, since individuals have easy access to PHC, due to universal coverage, very low co-payments, and wide geographical distribution, the GPs can easily reach children and youth.

Also, the PNSIJ includes guidelines for the evaluation of children and youths, indicating at which ages they must be evaluated and how. Eleven consultations are recommended between the first week and the third year of life, and eight consultations between three and 18 years old. The contents of the MH evaluation are clearly stated, mentioning affective relationships, behaviors and disorders, life at home, at childcare, and at school, substance abuse, violence, and physical abuse. Guidelines are widely available, but are poorly followed because of GPs’ lack of time, and also because the implementation of these guidelines is not clearly signalled and compensated. In practice, the evaluation of children is essentially centered on physical health, while adolescents often do not appear at these vigilance consultations.

#### Payment model

We suggest adding an indicator in the P4P scheme for PHC practices, namely the “percentage of users in the key-ages of the PNSIJ who have effectively attended the vigilance consultations, according to the diagnosis evaluation grid”.

Given that vigilance consultations are specific services that need to be encouraged, and that MH evaluation is more time consuming, we suggest the payment of an additional fee to GPs for each follow-up consultation including MH evaluation, using the diagnosis evaluation grid.

### Stepped collaborative model for depression

#### Rationale

According to WHO, “PHC is the main pillar supporting high-quality MH care” [6]. PHC has the capacity to identify and treat MH disorders, refer more severe cases to specialists, and carry out prevention and promotion activities. In particular, the treatment of common mental disorders by PHC services has several advantages over the treatment provided by specialized teams, in Portugal: (i) easier access related to the wide geographical distribution of PHC practices and the very low co-payments; (ii) holistic view of the patient, allowing the treatment of comorbidities; and (iii) a more efficient treatment, avoiding the use of more expensive specialized care.

#### Intervention

According to Gilbody et al. [27], there is strong evidence that the intervention of PHC in the treatment of depression is effective and cost-effective. We therefore opted to focus on this disease as a priority, which may be extended later to other MH diseases. The collaborative stepped care model has been demonstrated to be an effective response for the treatment of depression. Thirty-seven studies measured this effectiveness, showing improvements in terms of patient adherence to treatment, quality of life, and depression outcomes [28]. The model has been implemented differently in various places, namely in the UK [29], the Netherlands [30], the US [31], and Chile [32].

Based on the international experience, we suggest the implementation of a model in four stages:

*1st stage* Depression diagnosis in PHC, using a pre-defined symptoms grid (e.g., Patient Health Questionnaire, PHQ), by the GP or a nurse.

*2nd stage* Treatment of mild depression in PHC, on the basis of self-help, cognitive-behavioral therapy, and physical exercise, by a specialized MH worker.

*3rd stage* Treatment of moderate depression in PHC, on the basis of medication, psychological interventions, and social support, by the GP or psychologist.

*4th stage* Treatment of severe, atypical, or psychotic depression, or with suicide risk, on the basis of medication, complex psychological interventions, and combined treatments, by a specialized MH team including a psychiatrist.

The current payment scheme for PHC does not, however, provide incentives for their involvement in MH care. The capitation payment favors long patient lists, and thus leads to excess referral and overloading, and short consultations, which are not appropriate for MH therapies; finally, the P4P scheme does not include a single indicator related to MH.

#### Payment model

Following Miller, Ross [33], we propose the inclusion of the following indicator in the P4P scheme for PHC: “*Proportion of users with depression whose condition has been diagnosed with PHQ*-*9 and treatment has been initiated in the adequate phase of the collaborative stepped care model*”.

We also suggest nominating a reference GP in the PHC team and a reference psychiatrist in the specialized MH team of catchment area, to enhance the collaboration between primary and specialized care. We suggest the payment of a fixed monthly fee to compensate these physicians for the extra work. The availability of psychologists in PHC practices should also be considered.

### Integrated community-based care for SMI patients

#### Rationale

There is substantial evidence suggesting better outcomes for SMI when treated in the community, while inpatient stays are associated with poorer health outcomes and risk of readmissions [34]. Despite this evidence, there are few community-based MH teams in Portugal, while the current hospital financing model is volume-based, favoring more frequent consultations and inpatient stays.

#### Intervention

The model to be favored is that of community-based MH teams, which are expected to improve access to care because of their proximity to patients’ homes and lower stigma; to improve reinsertion because the community-based setting allows better contacts with social care, families, and employers; to improve follow-up, which leads to better health outcomes and efficiency through reducing inpatient stays and emergency visits.

#### Payment model

We suggest the implementation of a per period payment, according to which the hospital receives an annual payment for each patient registered with SMI, covering all healthcare services.

The rules for the payment attribution are the following:

- Diagnosed with SMI according to the International Classification of Diseases, 9th Revision, Clinical Modification (ICD-9-CM): 292 (drug-induced mental disorders), 295 (schizophrenic psychosis), 296 (affective psychosis), 297 (delirium illnesses), or 298 (non-organic psychosis).
- The number and type of patients are contracted at the beginning of the year, with the payment being attributed according to this estimated volume.
- The payment covers all SMI-related services, namely inpatient stays, day care, medications, consultations, lab tests, and exams.
- The payment does not cover the non-acute treatment phase, i.e., long-term care services.

Also, the participation in the new payment scheme is conditional on the following:

- The payment is attributed to the MH department, which has full autonomy and responsibility in managing funds, being the residual claimant.
- The MH department disposes of community-based MH teams, with protocols with PHC practices, residential units, patients and families associations, rehabilitation units, nursing homes, social services, and local authorities.

Also, the payment includes a P4P component:

- A bonus (penalty) for the hospitals in the lowest (highest) decile of the distribution in terms of inpatient stays.
- A bonus (penalty) for the hospitals in the lowest (highest) decile of the distribution in terms of post-discharge consultations up to 30 days after discharge.
- A budget penalty in case the hospital does not contribute and update a national registry of SMI, specifically created within this new payment model.

Finally, we suggest an implementation phase of this new payment scheme, in order to smooth the adaptation, collect new data, and evaluate its impact. The implementation will be limited to three hospitals in year 1, six hospitals in year 2, and nine hospitals in year 3.

The selection of hospitals for this pilot phase should be made using a random sampling method, from the universe of Portuguese NHS hospitals with a mental health department from the Lisbon, Coimbra, and Porto regions, where most patients are treated. We suggest selecting three hospitals used as “treatment group”, and three others as “control group”. Then, the same process will be replicated for the three following in year 2, and for the three last in year 3.

In their first implementation year, we suggest a 25% higher bundled payment, in order to favor the necessary changes in structures and teams. During the first 3 years, data will be collected on resource use, pathologies, and functionality, in order to refine the payment value and their risk-adjustment for functionality. Afterwards, the new payment model and its values will be designed, and the implementation will be extended to all the hospitals belonging to the Portuguese National Health Service (NHS).

## Discussion

This paper proposes an innovative payment model for the Portuguese public MH system. This system departs from the hypothesis that failures of previous plans, which have been largely highlighted in recent national and international evaluations [2, 7], are the result of the neglecting of implementation processes, especially in ensuring that suggested guidelines are properly financed and motivated. This is why in this project we focus on a payment model, as a means to implement best practices in MH.

Much has been written about the influence of payment models on healthcare providers’ practices [35, 36]. Surprisingly, only few studies have addressed the impact of reimbursement schemes in MH. This is why the proposal was mainly based on theoretical and empirical studies not specifically oriented toward MH, validated by MH experts. This resulted in the view that all payments have serious limitations, so that “payment innovations that blend elements of fee-for-service, capitation, and case rates can preserve the advantages and attenuate the disadvantages of each” [37] (p. 150). In other terms, it appears clearly that blended payments are the most promising option, combining several advantages of various payment schemes, in order to diminish their adverse effects. In the meantime, we selected the areas and types of interventions that best correspond to the current weaknesses of the Portuguese MH system, and for which there was more evidence.

This proposal needs to be tested in practice, to confirm whether the expected benefits will materialize in practice, and not be compromised by unexpected adverse effects. It should be highlighted that preliminary meetings have taken place at the Central Administration of the Health System (ACSS), the Portuguese institution that defines and implements the financing of NHS healthcare providers, in order to implement pilot projects following our recommendations. This is a promising step because these pilot projects include a close evaluation of their effectiveness and cost-effectiveness. Thus, we will be able, in the following months, to produce outcomes that we expect to be useful for Portugal and for other mental health systems facing similar difficulties.

### Limitations

Our proposal suffers from some limitations that should be mentioned. First, the proposal was presented to and validated by only a limited group of experts, selected by convenience. The choice of a convenience sample limited the representativeness of the people we interviewed. In particular, only five (out of 22) experts were from the North region, and none were from the (low-populated) Portuguese hinterland. Also, other professionals could have been interviewed, such as community workers, school teachers, or researchers in MH issues. If, as expected, the project creates interest in policy-makers for its implementation in practice, we suggest diffusing the proposal through formal channels, and opening a period for public discussion. Second, there is a vast literature on the effects of payment schemes on physicians’ practices, which inspired our model, but the literature is scarce on the empirical testing of their impact, and even much scarcer in the field of mental health. This is why we also suggest implementing the model progressively, in order to measure its effects carefully, before expanding it to the whole country. Finally, we must repeat that all payment schemes have their weaknesses, and even combining various models through blended formulas may not succeed in mitigating them. In particular, we propose to use in some way the pay-for-performance in all dimensions, which might be associated with excessive focus on incentivized indicators, crowding-out intrinsic motivation, or cheating on performance reporting [38]. Although the evidence is ambiguous for these adverse effects, they may be considered in the implementation process, through limiting the weight of the pay-for-performance in the physician remuneration.

### Budget impact and other implications

As our proposal is largely centered around implementing new financing mechanisms for MH providers, a major issue is its sustainability, in a country marked by a relatively low GDP per capita compared to other European countries, and tight public health budgets. Some of our suggestions are neutral from a budget viewpoint, as they merely redistribute money from low performers to high performers, in the case of pay-for-performance (dimensions 1, 2, and 3), or redistribute the money paid on the basis of volume into per-patient payments (dimension 4). However, in dimension 1 we propose a bundled payment to the PHC team for the follow-up of children at risk or with special needs during the first 2 years of life; in dimension 2, we suggest the payment of an additional fee to GPs for each follow-up consultation; and in dimension 3 the payment of a fixed monthly fee to compensate these physicians for the extra work, respectively. Considering an estimated number of 4722 children at risk and prices of each type of consultations, the annual budget impact of dimension 1 may vary between 1.3 and 2.4 million euros. Considering 1,964,862 children in the ages for the follow-up consultations, and a fee of 15 euros per consultation, the annual budget impact of dimension 2 would be of 29.5 million euros. Finally, considering the 857 primary care centers and 110 hospitals, and a monthly fee of 124 euros to GPs and specialists, the annual budget impact of dimension 3 would be of 1.2 million euros. In other terms, the budget impact of the proposal would be of 33.1 million euros per year, i.e., 0.36% of the total public health expenditures (9130 million euros in 2017).

Note, however, that providers’ payment mechanisms are only one among other possible instruments to promote best practices in MH, so that it should be accompanied by investments in community-based care facilities, continuous training and support for GPs, a greater autonomy for primary care and mental health department managers, and the reinforcement of primary care teams with psychologists. These investments also require an increasing awareness on the part of the population and decision-makers about the burden of MH disease, which financing models cannot achieve.

## Conclusion

The Portuguese MH system suffers from various weaknesses, and has failed to implement WHO recommendations on best practices. This failure is largely related to inadequate payment and incentives to providers. To overcome this problem, we designed an alternative payment model for primary care and hospitals on the basis of the literature and experts’ consultation. The model focuses on prevention and detection of diseases early in life, stepped care collaborative model for depression, and community-based care for SMI. This alternative financing model for mental healthcare providers, aimed at incentivizing best practices, is expected to contribute to a better quality of all MH financing systems that are facing the same challenges as the Portuguese one.

## Abbreviations

ACSS: Central Administration of the Health System
DRG: Diagnosis Related Group
DSM-IV: Diagnostic and Statistical Manual of Mental Disorders, 4th Edition
FFS: fee-for-service
GP: general practitioner
ICD-9-CM: International Classification of Diseases, 9th Revision, Clinical Modification
MH: mental health
NHS: National Health Service
PHC: primary healthcare
PHQ: Patient Health Questionnaire
PNSIJ: Portuguese National Plan for Child and Youth Health
P4P: pay-for-performance
SMI: serious mental illness
UK: United Kingdom
US: United States
WHO: World Health Organization
